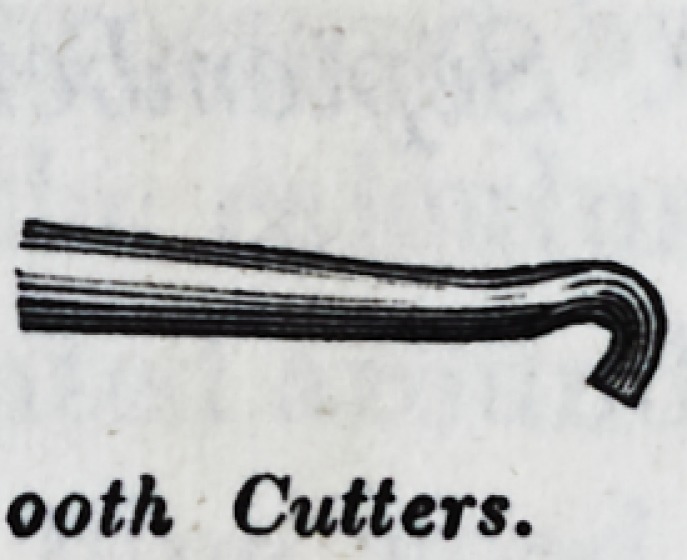# Coiled Gold for Plugging Teeth

**Published:** 1858-01

**Authors:** C. T. Cushman

**Affiliations:** Columbus, Ga.


### AMERICAN JOURNAL

OF

### DENTAL SCIENCE.

**Vol. VIII. NEW SERIES—JANUARY, 1858. No. 1.**

### ORIGINAL COMMUNICATIONS.

### ARTICLE I.

*Coiled Gold for Plugging Teeth.* By C. T. CUSHMAN, D. D. S., Columbus, Ga.

As this subject is beginning to attract some general interest, and seems to be misunderstood by many, I have thought proper to offer a few practical observations upon it, based upon ample experience.

I have used, daily, gold foil (and occasionally tin, platina and lead) in the form of ribbon coiled upon a winder, since the year 1847, in all cases where it was practicable—which, in my opinion, cannot include all the cases of an ordinary run of practice.

It is necessary to the success of this filling that the cavity have sides nearly at a right-angle with the bottom. These, together with a depth sufficiently proportioned to the circumference, cannot always be obtained; as for instance, in the side of a bicuspid, or of a front tooth. But where this form of gold *can* be used, it makes, in my opinion, the most perfect plug ;—possibly excepting Watts' late crystal gold, in cases where *that* is indicated. It is the most easily adapted to thin edged cavities with the least pressure, and where the cavity extends under the margin of the gum, it best holds the gum away from it, excludes moisture, and best insures perfection of density at that point, which, probably, is oftenest defective by other methods. Plugs thus made may restore the outline of the tooth-surface perfectly, and be in no possible danger of disintegration from the surface, as by other methods. Thus, a front incisor tooth laterally decayed away, the front and under plate of enamel, nearly as low as the bottom of the cavity, may be built up and rounded out by this means, and have its natural symmetry restored. Two such teeth adjoining, will in this way be rendered less conspicuous, and the space between them be much less than before filling. By the old method, such plugs are generally seen to be concave.

*Example of Space.*

In the grinding surfaces of teeth, the exact articulation with the opposing teeth may be restored; and where the back side, for instance, is broken out and gone, it may thus be built up, and the tooth rendered useful for a long time, by squaring down the sides and wedging these bolts or coils between.

In cases of decay on the front surface of a tooth—which are too common even among youth, and confessedly imperfect by the older plan of treatment—it is admirably adapted; the result resembling perfect *inlaying,* or mosaic work.

*Inlaid plug.*

In cases of decay on the inner side of the upper incisors, which are also common among youth, from hereditary imperfect formation, the operation of plugging, which in earlier times was confessedly difficult and performed with indifferent success, is now rendered comparatively easy and very secure, by a single coil.

A brittle, frail tooth, may be densely plugged in this way, with less force and less danger of fracture than by any other

I am acquainted with; the metal being in the most compact and best adapted form for solidification *before* being introduced into the cavity. For instance, I once treated an upper incisor, which having a defect near the centre of the front enamel, was neglected until the cavity would nearly contain a buck-shot, and extended quite to the inner plate of enamel. To pack this cavity from the bottom, with the other forms and method of gold, would assuredly have been to shiver it in pieces; but by this plan, the pressure being against the strongest portion of the tooth, and in its natural vertical direction; I plugged it solidly and successfully.

I cut a strip from a sheet of No. 4, a little wider than the depth of the cavity. The whole sheet was thus cut, and these continuously wound upon a fine broach, barely so tightly as to be still compressible. After obtaining the size which would just go within the opening, a sufficient end was left, with which to finish the vacancy made by compression. And here, at the first stage of the operation, I would stop and ask, in what way, and with so little labor, could so dense a body of gold be got into so small a space? I am satisfied, with my ten years' experience, that it is possible to make more solid plugs of a given bulk by this, than by any other known way. That pressure can make irregular masses of bulk pack within as small compass as smooth, even folds, may be asserted in tooth-filling, but certainly it cannot be demonstrated in your *carpet-bag*, although you literally "put your foot in it."

A ream of foolscap, if every sheet were opened, crumpled into balls, and twisted into ropes, could not, I believe, be thus compressed into their original-sized envelop, with the most resistless power known to mechanics. I do not, however, challenge, or seek controversy in this particular, as I have satisfied my own mind by years of careful experiment.

In a verbal discussion of practice, one remarked, in effect, that he did not understand how one managed to fill a tumbler with pipe-stems and compress them into a solid mass.

Others speak of winding them tightly, compressing them through a wire plate, then expanding them within the tooth. All this, I admit, is impracticable and not at all in accordance with my practice. Another has many "star-shaped cavities" to deal with, and thinks the "cylinders" must be illy adapted for such.

The grinding surfaces of the lower molars often have narrow crevices extending across them, but even these are most successfully plugged, in my opinion, on the coiled principle, the foil wound on a *flat* winder, (spatula shaped.) Such is my practice with these, because I find a narrow crevice is apt to become choked with gold introduced more in bulk.

Perhaps as much diversity of opinion has been exhibited concerning the best way of filling a tooth, as of any other mechanical operation. One recommends a multitude of minute pieces; another to cut the plug in pieces after it is solidified, for the mere purpose of having the cut surfaces unite more strongly than before ; just as some fresh amateurs *pulverize* a good violin, that the whole may be *improved* when the pieces are glued together! For me, I prefer a plug in a single piece, where that is practicable—and it often is—for the reason that I regard it as more perfect, and more secure than any other. This condition, however, is by no means absolute, for success and excellence.

In the use of coils (or cylinders) it is not necessary to *complete* the operation with them. It is often less practicable, as we reduce the space to be filled, than to unite the uncoiled end with the end of a *rope* of foil, and finish with that. Corners will be found too, with a sharp plugger, where some in this shape can best be forced in.

We all know that *small* cavities may be densely plugged with foil in various ways. Some of the finest plugs I have ever seen, were made with the rope twisted foil. This rope, however, must be light foil, and lightly twisted. A whole sheet of No. 4, thus treated, or wide-plaited, is sometimes the only practicable way of filling a desperate bicuspid, because of the great width and shallowness of the cavity.

For the coils, No. 4 is the best. The thicker numbers are more unmanageable when counter folded. For the same reason a whole sheet of No. 4 would not wind so well. This we have all familiarly illustrated to ourselves, in folding two sheets of paper into one letter form.

No. 6, in one-fourths, will generally fill, each, the anterior cavity in the grinding surface of the upper molars, without an additional piece. So of specific cavities, we may learn to know exactly what they require to fill them. The exact number of grains, when done is easily seen, and a note of this may prove useful for future reference. This has long been my system. During the entire excavation of the cavity, I am engaged in studying what form and quantity of gold will properly fill it; and am thus better prepared to decide that question.

*To make the Coils.*—I use a napkin folded square for the bed, and I press a straight edged paper-knife across the middle of the leaf, which depresses it, and raises the edges. With the same instrument I fold them together, and sometimes repeat the operation, to obtain the desired width, which should be a little more than the depth of the cavity to be filled. The plait is then wound upon a small broach, if the cavity be cylindrical, by holding the end between the thumb and finger; or upon a thin flat winder, of a width proportioned to its longest diameter, if oblong. I use three widths of the flat winder. A sheet of foil may be cut in fourths, eighths, or even sixteenths, for minute cavities, as on the front, or cutting edge of an incisor.

*Gold Coil Winders.*

Premising that the foil must necessarily be of the first quality, the following points should be observed in this style of operating.

1. Not to wind so tightly, but that the coil be left slightly impressible.

2. To make the coil a little longer than the depth of the cavity to be filled.

3. Not to pinch, break down, or mutilate the coil in introducing it.

4. To avoid letting it become displaced after it has received the first compression.

5. In all cylindrical and regular shaped cavities, to have the coil as large as will closely enter, and a free end over.

6. To fill to one side with an instrument as nearly as wide as the cavity as practicable, gradually exchanging, to suit the decreasing vacancy.

In the final, direct compression, a plugger covering the whole surface should be used; then smaller points.* File down, and repeat till solid; then polish.

For condensing plugs in the grinding surface of molars, I have for the last four years used, with great satisfaction, a right-angle plugger, having a lead covered shield for the opposing tooth to bite upon. With the handle of this firmly grasped in one hand, and its point steadied with the other, my patients, young and old, can exert a greater power, upon a hard plug than I can, by the force of their chewing muscles. Some persons have such *immense* strength in this way, that I have frequently to caution them against its over-exertion. A strong tooth might easily be split by such a contrivance. The force of these muscles has been estimated equal to the dead weight of the body. I believe it can be made to exceed this. It would be a critical test to apply to some large plugs I have seen, which their builders fondly regarded as "solid."

*** There are exceptions to this precept. I do not presume to teach—none need expect to learn—a manual art by written instructions.**

This instrument I apply to crystal gold plugs, also. A coiled gold foil plug, so treated, in a grinder of medium strength, has my entire confidence for solidity, and durability of wear. These coils after being so treated, may possibly be disintegrated, in the order they were put in; like plugs of tobacco from a keg. They do not inseparably unite, although they imperviously join.

*Self-acting Plug-condenser.*

A microscopist, in a recently published letter, having had occasion to prepare a thin section of tooth containing a plug, gives the dentist "a first rate notice" incidentally, in remarking that the plug was not dislodged by this treatment. If it were a coiled gold plug, the fact need excite no surprise.

I have had occasion to drill in on each side of such a plug of mine, which had been worn two years, and insert two others touching it, in the case of a clasp-tooth; then finished up the three together, which could not be distinguished from *one* original filling. The drilling obliquely towards the plug did not disturb it in the least.

I herewith submit samples of the coils, or bolts, (also called cylinders and blocks,) and also specimens of this kind of fillings, in various positions in the teeth, which I made in 1850.

In 1849 I so plugged the front surfaces of a young man's four upper incisors in this style; the cavities were large. They were pronounced by Dr. Bason, of North Carolina, the best he had ever seen. Compared with the old style of

*Cylindrical Gold Coils.*

*Flat Gold Coils.*

*stuffing,* which left a rough and porous surface, (a sample of which accompanies these,) the opinion was not extravagant. Another patient, who submitted this style of my fillings to Dr. C. A. Harris, in 1852, reported to me his saying he had never seen better.

The first mention of this form of foil for filling, that I remember, is by Lefoulon, in his "Noveau Traité Théorique et Pratique de l'art du Dentiste," p. 261—published in Paris, 1841. His preference, however, seems to have been for the plait without winding, which is really an excellent form for many cases, whether as a continuance of the coiled bolt, or by itself. The general principle of rolling, was more immediately urged upon my notice by an ingenious artizan, who was never in any way connected with dentistry. Being too easily satisfied however, with my then method of preparing and using foil, I did not fully adopt it till 1847.

My experience has suggested many improvements upon any ideas I had then obtained. For the suggestion of a *flat* winder, I am indebted to the late Dr. C. C. Allen of New York, in 1849. Plugs made in this way, he aptly remarked, were as solid as a block pavement. It is well adapted to the *roots* of teeth. A perfect *cone* can be wound, which will, by pressure covering its base, readily enter and adapt itself to the root of a tooth. Or, short cylindrical coils, of evenly graduated sizes, may be introduced; the smallest first, and thus filling as they go.

*Coiled foil.*

*Graduated cylindrical Coils.*

No way heretofore publicly proposed for plugging roots is equal to this in perfection of accomplishment.

If anticipating the possible necessity of removal however, I mostly use gutta percha in such cases.

For the outside of the first and second molars; the groove decay of the wisdom teeth; the largest cavities in the chewing surface of the grinders; those already specified, and others which will suggest themselves in practice, this form of metal offers, with the least labor, very satisfactory and durable results.

I opine that, eventually the graduated sized coils of a single ribbon will be accurately spun for, and sold in our market, to supply the daily demand of dentists.

*My process of treating lower molars extensively decayed on the grinding surface.*—These teeth, when the enamel is naturally defective—imperfectly crystallized, decay over more surface than any other, perhaps.

The most prevalent old practice was to plug the centre, and leave the radiating crevices to speedily undermine the plug. I invariably cut out the entire defect, with curved chisels and cutting excavators, making near a diamond shape cavity.

When the decay extends deep, and is very sensitive, the pulp not being exposed, I fill the bottom with Hill's gutta percha; making two distinct operations, and keeping both (as ever) dry, by the use of napkins. The upper part I then plug with coils (or crystal gold.) This practice, for several years, has been very successful and satisfactory with me. A tooth which was previously "tender," and even painful to cold, sweet, &c., may be thus made more comfortable than before, and very useful in chewing. It is not then liable to a new attack of decay on the same surface. The flooring being securely protected from wear, and from chemical action, serves as a perfect, and the most plastic, non-conductor with which I am acquainted. I can by this practice save many teeth which before treatment are useless, and ordinarily declined as impracticable, or extracted as incurable.

*Tooth Chisel.*

*ooth Cutters.*

While I hail with enthusiastic delight every advance toward perfecting the operation of plugging the teeth, I close by adding my convictions that no substance yet known, nor manner of using it, will infallibly save, or exempt them from further destruction.

A mechanical remedy is not a specific against a vital, or chemical effect.

The teeth may be destroyed by the fluids in which they exist.

Hereditary influences cannot be eradicated.

Health is no security against sickness.

Disease is innate and spontaneous.

We cannot mechanically provide for the teeth against the effect of fevers, gestation, dyspepsia, bronchitis, consumption, using tobacco, drinking spirits, &c.

It is unnatural to cut into the teeth, which are living bodies. If cut too deeply, they will *die,* as will a tree by like treatment. When dead they are brittle, and they will finally ulcerate at the root. For this, surgery has no cure.

The natural fate of the teeth is to crumble, and exfoliate, like the leaves of trees, in obedience to an irrevocable decree, the great law of nature.

*September* 1*st,* 1857.

## Figures and Tables

**Figure f1:**
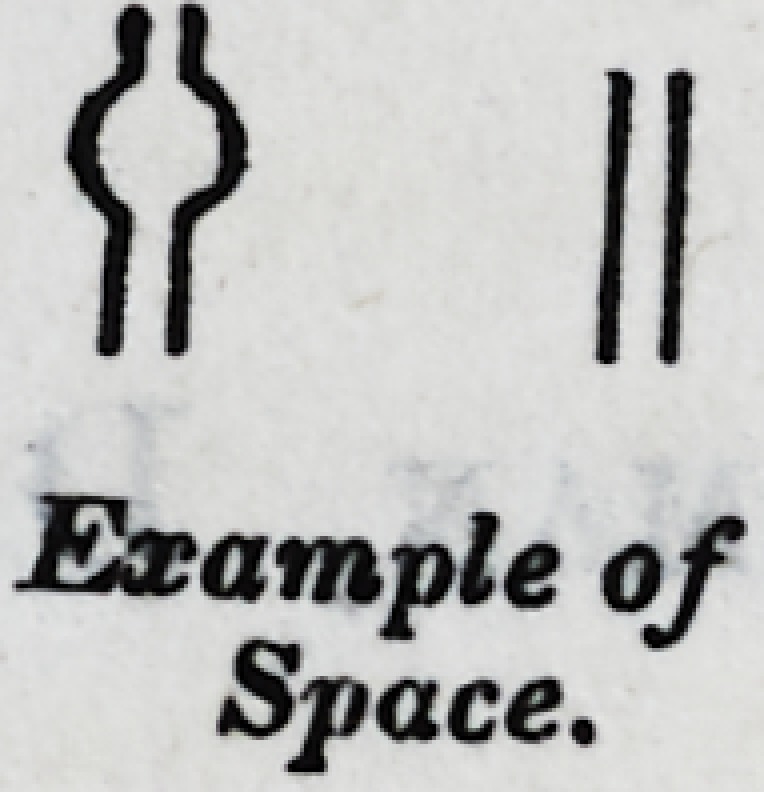


**Figure f2:**
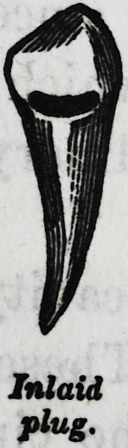


**Figure f3:**
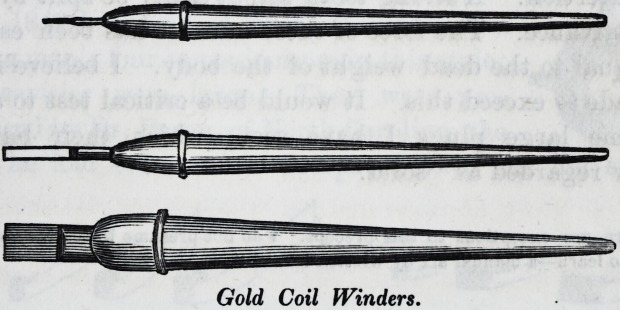


**Figure f4:**
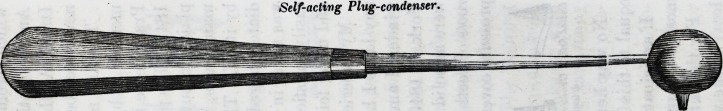


**Figure f5:**
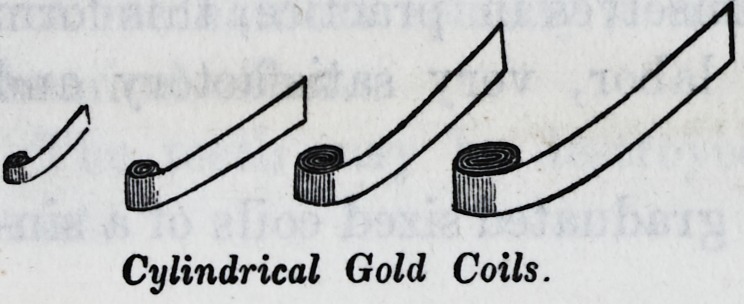


**Figure f6:**
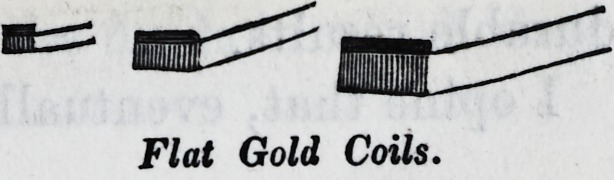


**Figure f7:**
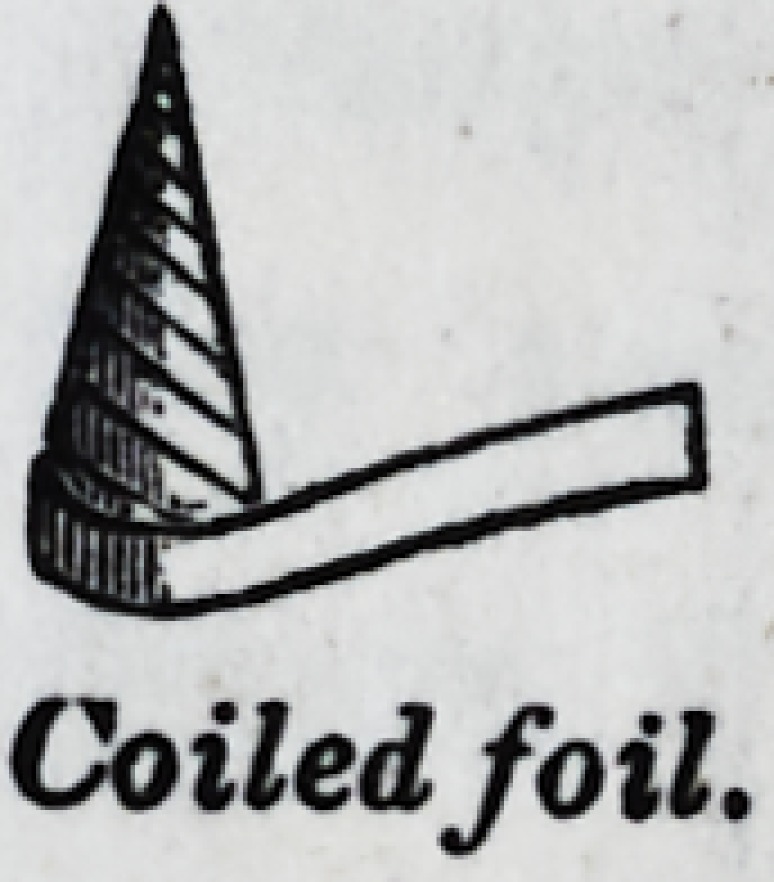


**Figure f8:**
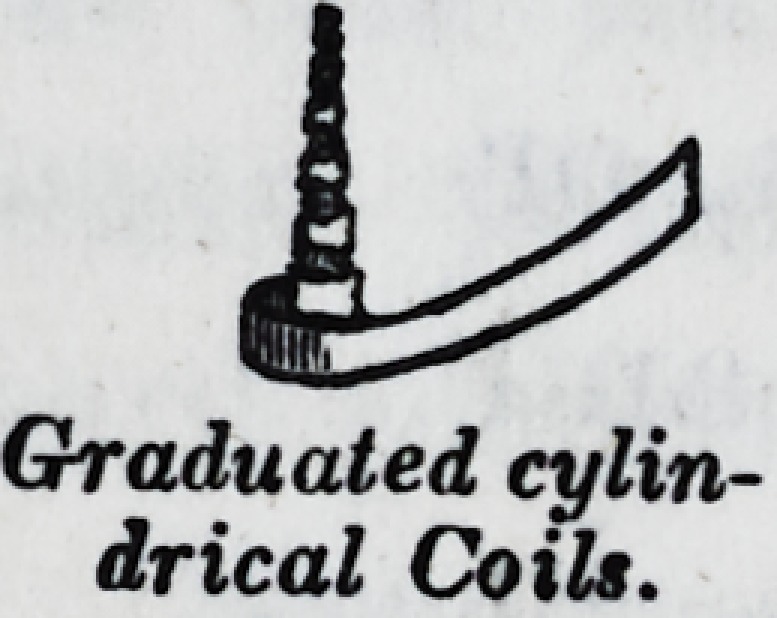


**Figure f9:**
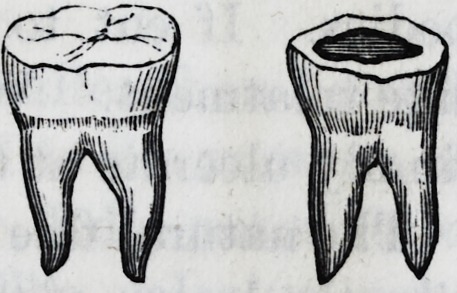


**Figure f10:**
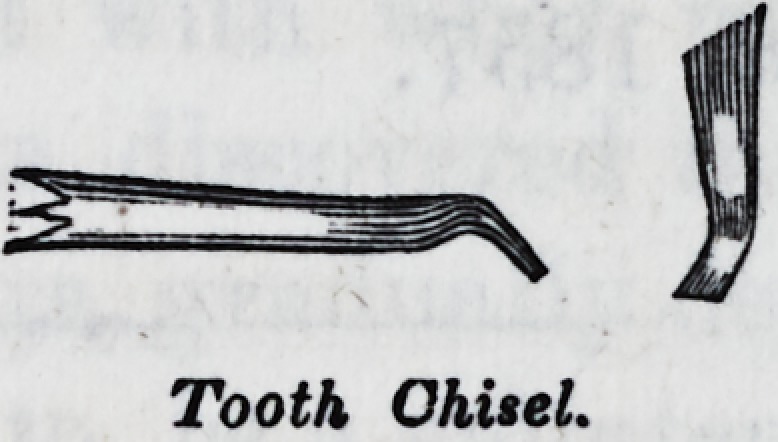


**Figure f11:**